# What the papers say

**DOI:** 10.1093/jhps/hny037

**Published:** 2018-10-30

**Authors:** Ajay Malviya

**Affiliations:** Northumbria Healthcare NHS Foundation Trust, Senior Lecturer, Regenerative Medicine - ICM, Newcastle University, 10 East Brunton Wynd, Newcastle upon Tyne, UK

The *Journal of Hip Preservation Surgery* (*JHPS*) is not the only place where work in the field of hip preservation may be published. Although our aim is to offer the best of the best, we continue to be fascinated by work that finds its way into journals other than our own. There is much to learn from it, so *JHPS* has selected six recent and topical subjects for those who seek a summary of what is taking place in our ever-fascinating world of hip preservation. What you see here are the mildly edited abstracts of the original articles to give them what *JHPS* hopes is a more readable feel. If you are pushed for time, what follows should take you no more than 10 min to read. So here goes. 

## HIP ARTHROSCOPY VERSUS BEST CONSERVATIVE CARE FOR THE TREATMENT OF FEMOROACETABULAR IMPINGEMENT SYNDROME (UK FASHIoN): A MULTICENTRE RANDOMIZED CONTROLLED TRIAL

High-level evidence to support the role of hip arthroscopy for impingement surgery is rapidly evolving. UK FASHIoN is a pragmatic, multicentre, assessor-blinded randomized controlled trial, done at 23 National Health Service hospitals in the UK [1]. Patients with femoroacetabular impingement (FAI) syndrome who presented at these hospitals were enrolled with clear inclusion and exclusion criteria. Patients with bilateral FAI syndrome were eligible; only the most symptomatic hip was randomly assigned to treatment and followed-up. Participants were randomly allocated (1:1) to receive hip arthroscopy or personalized hip therapy (an individualized, supervised and progressive physiotherapist-led programme of conservative care). Randomization was stratified by impingement type and recruiting centre and was done by research staff at each hospital, using a central telephone randomization service. Patients and treating clinicians were not masked to treatment allocation, but researchers who collected the outcome assessments and analysed the results were masked. The primary outcome was hip-related quality of life, as measured by the patient-reported International Hip Outcome Tool (iHOT-33) 12 months after randomization, and analysed in all eligible participants who were allocated to treatment (the intention-to-treat population). 

Between 20 July 2012 and 15 July 2016, 648 eligible patients were identified and 348 were recruited: 171 participants were allocated to receive hip arthroscopy and 177 to receive personalized hip therapy. Three further patients were excluded from the trial after randomization because they did not meet the eligibility criteria. Follow-up at the primary outcome assessment was 92% (319 of 348 participants). At 12 months after randomization, mean iHOT-33 scores had improved from 39.2 to 58.8 for participants in the hip arthroscopy group, and from 35.6 to 49.7 in the personalized hip therapy group. In the primary analysis, the mean difference in iHOT-33 scores, adjusted for impingement type, sex, baseline iHOT-33 score, and centre, was 6.8 in favour of hip arthroscopy (*P* = 0.0093). This estimate of treatment effect exceeded the minimum clinically important difference (6.1 points). There were 147 patient-reported adverse events [in 100 (72%) of 138 patients] in the hip arthroscopy group) versus 102 events [in 88 (60%) of 146 patients] in the personalized hip therapy group, with muscle soreness being the most common of these [58 (42%) versus 69 (47%)]. There were seven serious adverse events reported by participating hospitals. Five (83%) of six serious adverse events in the hip arthroscopy group were related to treatment, and the one in the personalized hip therapy group was not. There were no treatment-related deaths, but one patient in the hip arthroscopy group developed a hip joint infection after surgery.

In a larger multicentred randomized controlled setting it has been now demonstrated that hip arthroscopy and personalized hip therapy both improved hip-related quality of life for patients with FAI syndrome; hip arthroscopy led to a greater improvement than did personalized hip therapy, and this difference was clinically significant. The authors concluded that further follow-up will be required to identify whether the clinical benefits of hip arthroscopy are maintained and whether it is cost effective in the long term.

## DANISH HIP ARTHROSCOPY REGISTRY: PREDICTORS OF OUTCOME IN PATIENTS WITH FAI

Registry data have certain advantages over studies published from select centres or a systematic review of those studies; because it includes data of surgeons with varying skill levels, who may not be producing volumes that would be individually published and in a way gives a voice to the ‘silent’ minority that probably do the majority of these procedures.

The Danish Hip Arthroscopy Registry (DHAR) is a great template to be adopted. In this prospective study from the DHAR, the authors set out to identify predictors of poor outcome after FAI surgery [2]. The primary hypothesis was that older patients, patients with severe cartilage damage and female patients might have inferior outcome results compared with younger patients, patients with minor cartilage damage and male patients.

Radiological and surgical data as well as patient-reported outcome measures (PROM) from FAI patients in DHAR between January 2012 and May 2015 were collected. PROMs consisting of Copenhagen Hip and Groin Outcome Score (HAGOS), quality of life (EQ-5D), Hip Sports Activity Scale (HSAS) and Numeric Rating Scale (NRS) pain scores were assessed. The patients were divided into three age groups (<25, 25–39 and ≥40 years). Cartilage injuries were classified according to International Cartilage Repair Society (ICRS) (femoral side) and modified Becks (acetabular side) classifications. A non-parametric statistic method was used to analyse the differences between the pre- and post-operative PROM values.

Data from 2054 FAI procedures in DHAR were collected. Fifty-three percent of the procedures were done in female patients. All HAGOS sub-scales, EQ-5D, HSAS and NRS pain (rest and walk) demonstrated significant improvements in all age groups at follow-up. Comparison between age groups demonstrated poorer outcomes in both older age groups when compared with the <25 years age group at 1- and 2-year follow-ups. Higher degrees of femoral and acetabular cartilage injury did have a negative influence on outcome at follow-up. Comparison between genders demonstrated lower pre-operative outcomes in females and lower outcome score (HSAS) 1 and 2 years after FAI surgery.

The authors confirmed that age above 25 and major cartilage injury might negatively affect the outcome of surgery, however, gender could not be identified as a negative predictor of clinical outcome after FAI surgery, but might negatively affect sports participation in females.

## TIME REQUIRED TO ACHIEVE MINIMAL CLINICALLY IMPORTANT DIFFERENCE AND SUBSTANTIAL CLINICAL BENEFIT AFTER ARTHROSCOPIC TREATMENT OF FAI

The primary goal of surgery is to obtain a substantial improvement in the patients’ symptoms to allow them to reach their best possible activity level. Minimal clinically important difference (MCID) defines the minimum degree of quantifiable outcome improvement that a patient perceives as the result of an intervention or in the process of healing. Substantial clinical benefit (SCB) defines the amount of quantifiable outcome improvement that is needed for a patient to feel substantially better. Nwachukwu *et al*. [3] from the Hospital for Special Surgery, New York, found that little is known about when clinically significant outcome improvement is achieved and have prospectively investigated the time-dependent nature of MCID and SCB after hip arthroscopy for FAI.

The institutional hip preservation registry was queried which used the modified Harris Hip Score, Hip Outcome Score, and 33-item International Hip Outcome Tool (iHOT-33) for patients undergoing hip arthroscopy for FAI. Follow-up times for outcome measures were classified into three periods: 5–11 months (6 months), 12–23 months (1 year) and 24–35 months (2 years). Cumulative probabilities for achieving MCID and SCB were calculated with Kaplan–Meier survival curve analysis and interval censoring. A Weibull parametric regression analysis evaluated the odds of achieving earlier MCID.

A total of 719 patients undergoing primary hip arthroscopy were included. The mean age was 32.5 years with a female preponderance (52.9%). Across all four outcome instruments, patients had the highest probability for achieving MCID and SCB by the 6-month post-operative period. The iHOT-33 demonstrated the highest probability for capturing MCID and SCB improvement at each of the three periods, with 76.0, 84.8 and 93.6% achieving MCID by 6 months, 1 and 2 years, respectively. Similarly, the probabilities of achieving SCB on the iHOT-33 were as follows: 57.1, 68.0, and 71.7%. A similar trend was demonstrated across other outcome tools. Older male patients and those with Outerbridge classification 1–4 (versus grade 0) had a significantly increased risk for taking a longer time to achieve MCID and SCB. Additionally, patients with higher pre-operative outcome scores took a longer time to achieve MCID and SCB.

The study has concluded that at least half of patients treated with hip arthroscopy for FAI achieve MCID and SCB within the first 6 months after the procedure. However, clinically significant outcome improvement continues to be attained until 2 years post-operatively. Female patients, younger individuals, and those without chondral defects achieve faster clinical outcome improvement.

## CAN WE DEVELOP A PREDICTIVE SCORE FOR OUTCOME AFTER ARTHROSCOPIC SURGERY FOR FAI?

Italian researchers [4] set out to build a post-arthroscopy outcome-predictive score (POPS) that could be associated with the likelihood of lasting benefit after arthroscopic treatment of FAI and based solely on unambiguous pre-operative information.

A population of 220 FAI patients, operated on with standard techniques by orthopaedic surgeons trained in hip arthroscopy in six different Italian centres, was evaluated physically or by telephone interview 2–5 years after surgery. The criteria of successful mid-term outcome (SMO) were agreed upon by all authors. A multivariate logistic regression, adjusted for patient's age and centre, was applied to predict SMO. In the model, the variables associated with the outcome were included and the relative odds ratios (ORs) were used to compute the FAI-POPS (FAI—POPS). A receiver operating characteristic curve was plotted and the optimum cut-off was calculated.

Totally, 155 patients out of 220 showed a successful mid-term outcome. The optimum cut-off of FAI-POPS was calculated to be 6.3 and with this threshold it proved a sensitivity of 0.66 and a specificity of 0.69, a positive predictive value of 0.84 and a negative predictive value of 0.46. The standard arthroscopic treatment of FAI resulted in satisfactory persistent symptom relief for about 70% of patients. No or minimal osteoarthritis, short time elapsed from the appearance of symptoms and high pre-operative modified Harris Hip Score are independent predictive factors of SMO.

The authors concluded that FAI-POPS, obtained as sum of three ORs corresponding to the above prognostic factors, is a useful predictor of mid-term outcome after conventional arthroscopic FAI treatment.

## DOES ANTERIOR ACETABULAR COVERAGE INFLUENCE THE CLINICAL OUTCOME OF ARTHROSCOPICALLY TREATED CAM-TYPE FAI?

What represents clinically significant acetabular undercoverage in patients with symptomatic cam-type FAI remains controversial. The aim of this study from Ottawa [5] was to examine the influence of the degree of acetabular coverage on the functional outcome of patients treated arthroscopically for cam-type FAI.

The authors identified 88 patients (97 hips) who underwent arthroscopic cam resection and concomitant labral debridement and/or refixation. There were 57 male and 31 female patients with a mean age of 31.0 years and a mean body mass index of 25.4 kg m^−^^2^.

The Hip2Norm, an object-oriented-platform program, was used to perform 3D analysis of hip joint morphology using 2D anteroposterior pelvic radiographs. The lateral centre-edge angle, anterior coverage, posterior coverage, total femoral coverage, and alpha angle were measured for each hip. The presence or absence of crossover sign, posterior wall sign, and the value of acetabular retroversion index were identified automatically by Hip2Norm. Patient-reported outcome scores were collected pre-operatively and at final follow-up with the Hip Disability and Osteoarthritis Outcome Score (HOOS).

At a mean follow-up of 2.7 years, all functional outcome scores significantly improved overall. Radiographically, only pre-operative anterior coverage had a negative correlation with the improvement of the HOOS symptom subscale (*r* =−0.28, *P* = 0.005). No significant difference in relative change in HOOS subscale scores was found according to the presence or absence of radiographic signs of retroversion.

The study has demonstrated that anterior coverage is an important modifier influencing the functional outcome of arthroscopically treated cam-type FAI.

## CAN T2 MAPPING MRI BE USED TO EVALUATE ARTICULAR CARTILAGE TO DETERMINE SUCCESS OF HIP PRESERVING SURGERY?

Shoji *et al*. [6] from Hiroshima University, Japan aimed to evaluate the post-operative changes of articular cartilage and whether the pre-operative condition of the articular cartilage influences the clinical results using T2 mapping MRI.

They reviewed 31 hips with early stage osteoarthritis in 31 patients (mean age, 39.6 years), including three men and 28 women who underwent rotational acetabular osteotomy (RAO) for hip dysplasia. Clinical evaluations including Japanese Orthopaedic Association (JOA) score and Japanese Orthopaedic Association Hip Disease Evaluation Questionnaire (JHEQ) and radiographical evaluations on X-ray were performed. Longitudinal qualitative assessment of articular cartilage was also performed using 3.0-T MRI with T2 mapping technique pre-operatively, 6 months, and at 1 and 2 years post-operatively.

There was no case with progression of osteoarthritis. The mean JOA score improved from 70.1–93.4 points, the mean post-operative JHEQ score was 68.8 points, and radiographical data also improved post-operatively. They reported that the T2 values of the cartilage at both femoral head and acetabulum increased at 6 months on coronal and sagittal views. However, they significantly decreased 1 and 2 years post-operatively. The T2 values of the centre to anterolateral region of acetabulum negatively correlated with post-operative JHEQ score, particularly in pain score.

This study suggests that biomechanical and anatomical changes could apparently cause decreased T2 values 1–2 years post-operatively compared with those pre-operatively. Furthermore, pre-operative T2 values of the acetabulum can be prognostic factors for the clinical results of RAO.

## CONFLICT OF INTEREST STATEMENT

None declared.
